# N-acetyl-L-leucine for Niemann-Pick type C: a multinational double-blind randomized placebo-controlled crossover study

**DOI:** 10.1186/s13063-023-07399-6

**Published:** 2023-05-29

**Authors:** T Fields, T M. Bremova, I Billington, GC Churchill, W Evans, C Fields, A Galione, R Kay, T Mathieson, K Martakis, M Patterson, F Platt, M Factor, M Strupp

**Affiliations:** 1 IntraBio Ltd, Begbroke Science Park, Begroke Hill, Woodstock Road, Oxford, OX5 1PF UK; 2 Department of Neurology, Inselspital, University Hospital Bern, and University of Bern, Bern, Switzerland; 3 Department of Pharmacology, University of Oxford, Mansfield Road, Oxford, OX1 3QT UK; 4 Niemann-Pick UK, Suite 2, Vermont House, Concord, Tyne and Wear, Washington, NE37 2SQ UK; 5 Primary Care Stratified Medicine (PRISM), Division of Primary Care, University of Nottingham, Nottingham, UK; 6 RK Statistics, Brook House, Mesne Lane, Bakewell, DE45 1AL UK; 7 Department of Pediatric Neurology, University Children’s Hospital (UKGM) and Medical Faculty, Justus Liebig University of Giessen, Giessen, Germany; 8 Mayo Clinic, 200 First Street SW, Rochester, MN 55905 USA; 9 Department of Neurology and German Center for Vertigo and Balance Disorders, Ludwig Maximilians University, Munich, Germany

**Keywords:** Niemann-Pick type C (NPC), N-acetyl-L-leucine, Pharmaceutical intervention, Symptomatic treatment, Randomized controlled trial, Cerebellar ataxia, Lysosomal storage disease

## Abstract

**Background:**

Niemann-Pick disease type C (NPC) is a rare autosomal recessive neurodegenerative lysosomal disease characterized by multiple symptoms such as progressive cerebellar ataxia and cognitive decline. The modified amino acid N-acetyl-leucine has been associated with positive symptomatic and neuroprotective, disease-modifying effects in various studies, including animal models of NPC, observational clinical case studies, and a multinational, rater-blinded phase IIb clinical trial. Here, we describe the development of a study protocol (Sponsor Code “IB1001-301”) for the chronic treatment of symptoms in adult and pediatric patients with NPC.

**Methods:**

This multinational double-blind randomized placebo-controlled crossover phase III study will enroll patients with a genetically confirmed diagnosis of NPC patients aged 4 years and older across 16 trial sites. Patients are assessed during a baseline period and then randomized (1:1) to one of two treatment sequences: IB1001 followed by placebo or vice versa. Each sequence consists of a 12-week treatment period. The primary efficacy endpoint is based on the Scale for the Assessment and Rating of Ataxia, and secondary outcomes include cerebellar functional rating scales, clinical global impression, and quality of life assessments.

**Discussion:**

Pre-clinical as well as observational and phase IIb clinical trials have previously demonstrated that IB1001 rapidly improved symptoms, functioning, and quality of life for pediatric and adult NPC patients and is safe and well tolerated. In this placebo-controlled cross-over trial, the risk/benefit profile of IB1001 for NPC will be evaluated. It will also give information about the applicability of IB1001 as a therapeutic paradigm for other rare and common neurological disorders.

**Trial registrations:**

The trial (IB1001-301) has been registered at www.clinicaltrials.gov (NCT05163288) and www.clinicaltrialsregister.eu (EudraCT: 2021–005356-10). Registered on 20 December 2021.

**Supplementary Information:**

The online version contains supplementary material available at 10.1186/s13063-023-07399-6.

## Introduction

### Background

Niemann-Pick disease type C (NPC) is a rare (1:120,000 live births), prematurely fatal, autosomal recessive, neurovisceral lysosomal disease that predominantly affects children. The disease typically begins in early childhood, is chronic, progressive, and severely reduces quality of life. Adolescent and adult-onset cases are being increasingly recognized [1]. The presentation of NPC is characterized by broad heterogeneity in systemic, psychiatric, and neurological symptoms, though in general, the earlier the age of onset, the more rapidly progressive the symptoms [2]. There is broad inter-individual phenotypic variability, including the age of onset and rate of progression. This renders an assembly of well-matched cohorts of NPC patients for controlled trials difficult to achieve. Treatment of NPC is currently limited to reducing the rate of disease progression with the substrate reduction therapy drug miglustat (Zavesca™), which is approved in the European Union and several other countries, but not in the USA [3].

### N-acetyl-L-leucine

N-acetyl-L-leucine (Sponsor Code “IB1001”) is the L-enantiomer of N-acetyl-DL-leucine, a modified, acetylated derivative of a natural essential amino acid (Leucine) that has been available in France since 1957 as a racemate (equal amounts of both D- and L-enantiomers) under the trade name Tanganil™ (Pierre Fabre Laboratories, France) as a treatment for acute vertigo. Prior observational studies assessing the effect of N-acetyl-DL-leucine in patients with NPC suggest a beneficial symptomatic as well as neuroprotective disease-modifying effect of this agent. In a case series, short-term treatment with N-acetyl-DL-leucine was found to improve ataxia, cognition, and quality of life in 12 patients with NPC [4]. Subsequent long-term case series and pre-clinical studies demonstrated the neuroprotective, disease-modifying effect of treatment in NPC [5, 6]. In all studies, the compound was well tolerated with no serious side effects.

Recently, two multinational, phase IIb clinical trials with N-acetyl-L-leucine for NPC and the related lysosomal storage disorder GM2 gangliosidosis (Tay-Sachs and Sandhoff diseases) were completed [7–9]. Both trials were successful, demonstrating a statistically significant change on the primary and secondary endpoints and clinically meaningful improvement in symptoms, functioning, and quality of life for children and adults with NPC and GM2. In both studies, N-acetyl-L-leucine was also well tolerated with no serious side effects.

Animal studies in the NPC mouse model have shown that the L-enantiomer, i.e., N-acetyl-L-leucine, has potential clinical benefits compared to the racemic mixture. N-acetyl-L-leucine is a pro-drug of L-leucine and has a unique transport mechanism [10]. One mechanism of action of N-acetyl-L-leucine is the activation of cerebral glucose metabolism in the cerebellum, correlated with enhanced cerebellar activity [11, 12]. In an animal model of NPC, N-acetyl-DL-leucine and its enantiomers significantly reduced ataxia in *Npc1*^*−/−*^ mice, when treated symptomatically (from 8 to 9 weeks of age) and pre-symptomatically (from 3 weeks of age) [6]. These studies specifically identified the L-enantiomer as the neuroprotective isomer*,* observed to significantly delay the onset of functional decline (gait abnormalities, motor dysfunction), the decline in general health and condition, as well as slowing disease progression and prolonging survival (whereas the D-enantiomer did not). Similar effects of N-acetyl-L-leucine were found in an animal model of another lysosomal storage disease, the Sandhoff (*hexb*^*−/−*^*)* mouse [13]. It is important to note that the dosage used in these in vivo studies (0.1 g per kg per day) approximates the dose used in previous observational clinical studies with the racemate, the phase IIb studies with IB1001, and the current IB1001-301 clinical trial. Finally, pharmacokinetic studies demonstrate that the D-enantiomer is not metabolized and could accumulate relative to the L-enantiomer during chronic administration of the racemate, having the potential for long-term negative effects [14]. Therefore, in this clinical trial, the effects of N-acetyl-L-leucine will be evaluated.

### Trial rationale

The primary objective of the IB1001-301 clinical trial is to demonstrate the symptomatic benefits of IB1001 treatment given:

(i) There are no treatments for the symptoms of NPC, which are highly debilitating and significantly detrimental to functioning, quality of life, and health span.
(ii) NPC is a life-limiting condition with an extremely high unmet medical need, mandating greater urgency for trials to be conducted as efficiently as possible to maximize the chance they can be made available before the window of therapeutic opportunity is lost [8].

Accordingly, although there is data supporting both the symptomatic and neuroprotective, disease-modifying effects of IB1001 for NPC, the IB1001-301 pivotal trial prioritizes the investigation of the symptomatic benefit to expedite the development and availability of this promising drug candidate. Given the extreme heterogeneity of the disease, a crossover study design is utilized so that each patient serves as their own control and the clinical meaningfulness of the effect can be assessed for each patient.

## Methods/design

### Study oversight

The IB1001-301 trial is conducted in accordance with the International Conference for Harmonisation (of Technical Requirements for Pharmaceuticals for Human Use)—Good Clinical Practice Guideline, the General Data Protection Regulator, and the Declaration of Helsinki. The study protocol was designed in accordance with the SPIRIT 2013 statement and the study is conducted in accordance with the SPIRIT reporting guidelines [15]; the SPIRIT Figure for both the Parent Study and the Extension Phase is included as Supplementary Tables 1 and 2, and the SPIRIT checklist has been completed. The study has been approved by the ethics committees of each participating center and the regulatory authorities in each respective country. The safety, integrity, and feasibility of the trial is monitored by an independent data safety monitoring board (DSMB) consisting of three independent, non-participating members (including two clinicians and a statistician). The function of the DSMB is to monitor the course of the studies and, as applicable, recommend to the sponsor of the trial for discontinuation, modification, or continuation of the study. The roles and responsibilities of the DSMB are defined in a DSMB charter.

### Patient population and eligibility criteria

Patients will be screened for eligibility according to the inclusion and exclusion criteria. To be eligible for the respective study, patients aged ≥ 4 years with a confirmed diagnosis of NPC must present with clinical symptoms, provide appropriate informed consent, and undertake a washout of any prohibited medications (if applicable). These include any variant of N-acetyl-DL-leucine (e.g., Tanganil™). For a detailed description of the inclusion and exclusion criteria, see Table 1.

**Table 1:** Inclusion and exclusion criteria for patient selection in the Parent Study

Inclusion criteria	Exclusion criteria
Individuals who meet all of the following criteria are eligible to participate in the study: 1. Written informed consent signed by the patient and/or their legal representative/parent/impartial witness 2. ***Non-US:*** Male or female aged ≥ 4 years with a confirmed diagnosis of NPC at the time of signing informed consent. Confirmed diagnosis includes one of the following: a) Clinical features and positive biomarker screen and/or filipin test without genetic tests results (has not been performed) b) Clinical features and positive genetic test c) Clinical features and positive biomarker screen and/or filipin test but only one NPC mutation identified on genetic test d) Clinical features with positive biomarker screen and/or filipin test and positive genetic test *** US:*** Male or female aged ≥ 4 years with a confirmed genetic diagnosis of NPC at the time of signing informed consent. Patients must have clinical features of NPC and a positive genetic test for mutations in both copies of NPC1 or in both copies of NPC2 3. Females of childbearing potential, defined as a premenopausal female capable of becoming pregnant, will be included if they are either sexually inactive (sexually abstinent^a^ for 14 days prior to the first dose and confirm to continue through 28 days after the last dose) or using one of the following highly effective contraceptives (i.e., results in < 1% failure rate when used consistently and correctly) 14 days prior to the first dose continuing through 28 days after the dose: a) Intrauterine device (IUD); b) Surgical sterilization of the partner (vasectomy for 6 months minimum); c) Combined (estrogen or progestogen containing) hormonal contraception associated with the inhibition of ovulation (either oral, intravaginal, or transdermal); d) Progestogen only hormonal contraception associated with the inhibition of ovulation (either oral, injectable, or implantable); e) Intrauterine hormone releasing system (IUS); f) Bilateral tubal occlusion 4. Females of non-childbearing potential who have undergone one of the following sterilization procedures at least 6 months prior to the first dose: a) Hysteroscopic sterilization; b) Bilateral salpingectomy; c) Hysterectomy; d) Bilateral oophorectomy; ** OR** be postmenopausal with amenorrhea for at least 1 year prior to the first dose and follicle stimulating hormone (FSH) serum levels consistent with postmenopausal status. FSH analysis for postmenopausal women will be done at screening. FSH levels should be in the postmenopausal range as determined by the central laboratory 5. Non-vasectomized male patient agrees to use a condom with spermicide or abstain from sexual intercourse during the study until 90 days beyond the last dose of study medication and the female partner agrees to comply with inclusion criteria 3 or 4. For a vasectomized male who has had his vasectomy 6 months or more prior to study start, it is required that they use a condom during sexual intercourse. A male who has been vasectomized less than 6 months prior to study start must follow the same restrictions as a non-vasectomized male 6. If male, patient agrees not to donate sperm from the first dose until 90 days after their last dose 7. Patients must fall within: a) A SARA score of 7 ≤ X ≤ 34 points (out of 40) ** AND** b) Either: i. Within the 2–7 range (0–8 range) of the Gait subtest of the SARA scale ** OR** ii. Be able to perform the 9-Hole Peg Test with Dominant Hand (9HPT-D) (SCAFI subtest) in 20 ≤ X ≤ 150 s 8. Weight ≥ 15 kg at screening 9. Patients are willing to disclose their existing medications/therapies for (the symptoms) of NPC, including those on the prohibited medication list. Non-prohibited medications/therapies (authorized medicines for NPC [e.g., miglustat], speech therapy, and physiotherapy are permitted provided: a) The investigator does not believe the medication/therapy will interfere with the study protocol/results b) Patients have been on a stable dose/duration and type of therapy for at least 42 days before **visit 1** (baseline 1) c) Patients are willing to maintain a stable dose/do not change their therapy throughout the duration of the study 10. An understanding of the implications of study participation, provided in the written patient information and informed consent by patients or their legal representative/parent, and demonstrates a willingness to comply with instructions and attend required study visits (for children this criterion will also be assessed in parents or appointed guardians)	Individuals who meet any of the following criteria are not eligible to participate in the study: 1. Patients who have any known hypersensitivity or history of hypersensitivity to: a. Acetyl-leucine (DL-, L-, D-) or derivatives b. Excipients the IB1001 sachet (namely isomalt, hypromellose, and strawberry flavor) c. Excipients the placebo sachet (namely isomalt, hypromellose, strawberry flavor, citric acid, microcrystalline cellulose, lactose, denatonium benzoate) 2. Simultaneous participation in another clinical study or participation in any clinical study involving administration of an investigational medicinal product (IMP; “Study drug”) for at least 42 days prior to **visit 1**. At the discretion of the investigator, medical monitor, and sponsor, the washout period for specific IMPs may be longer based on the pharmacological activity and pharmacokinetics of the drug 3. Patients with a physical or psychiatric condition which, at the investigator’s discretion and in consultation with the medical monitor and sponsor (as applicable), may put the patient at risk, may confound the study results, or may interfere with the patient’s participation in the clinical study, i.e., reliably perform study assessments 4. Known or persistent use, misuse, or dependency of medication, drugs, or alcohol 5. Current or planned pregnancy or women who are breastfeeding 6. Patients with severe vision or hearing impairment (that is not corrected by glasses or hearing aids) that, at the investigator’s discretion, interferes with their ability to perform study assessments 7. Patients who have been diagnosed with arthritis or other musculoskeletal disorders affecting joints, muscles, ligaments, and/or nerves that by themselves affects patient’s mobility and, at the investigator’s discretion, interferes with their ability to perform study assessments 8. Patients unwilling and/or not able to undergo a 42-day washout period from any of the following prohibited medication prior to **visit 1** (baseline 1) and remain without prohibited medication through **visit 6** a) N-acetyl-DL-leucine (e.g., Tanganil®); b) N-acetyl-L-leucine (prohibited if not provided as IMP in the IB1001-301 trial); c) Sulfasalazine; d) Rosuvastatin

^a^Sexual abstinence is considered a highly effective method only if defined as refraining from heterosexual intercourse during the entire period of risk associated with the study treatments. In this trial abstinence is only acceptable if in line with the patient’s preferred and usual lifestyle. Period abstinence (calendar, symptothermal, post-ovulation methods), withdrawal (coitus interruptus), spermicides only, and lactational amenorrhoea method (LAM) are not acceptable methods of contraception. As well, female condom and male condom should not be used together

### Recruitment and patient involvement

The principal investigator at each site will be responsible for the enrolment of patients. Patients will be screened at 16 centers across Australia, the Czech Republic, Germany, the Netherlands, Slovakia, Switzerland, the UK, and the USA. The list of study sites is available via www.clinicaltrials.gov (NCT05163288). Patients will be recruited via personal correspondence, routine care appointments, and referrals. In addition, there is collaboration and support from multinational patient organizations representing these rare disease communities. All eligible patients who agree to participate in the study are provided with a full verbal explanation of the trial and the Patient Information Sheet. This includes detailed information about the rationale, design, and personal implications of the study.

### Study design and procedures

The IB1001-301 clinical trial is a double-blind placebo-controlled crossover trial. Patients will be first assessed during a baseline period (with or without a study run-in) and randomized (1:1) to one of two treatment sequences: IB1001 followed by placebo, or vice-versa. Each treatment period will last approximately 12 weeks (84–91 days). Patients will be assessed twice during each period to allow an assessment of intra-patient variability.

#### Screening

At the initial screening visit, patients will be classified as either “naïve” or “non-naïve” depending on their use of prohibited medications within the past 6 weeks (42 days). The schedule of events during the initial screening visit and throughout the baseline period (through visit 1) will vary depending on the patient’s classification as either “naïve” or “non-naïve.” Given the known unlicensed use of the racemate (Tanganil™), for all patients, a urine sample will be taken at visit 1 to detect N-acetyl-D-leucine using a validated liquid chromatography mass spectrometry/mass spectrometry method. Provided the level of N-acetyl-D-leucine is below the permitted threshold, the initial screening visit will be confirmed as visit 1 (baseline 1). If a patient classified as “Naïve” unexpectedly tests positive for levels of N-acetyl-D-leucine above the permitted threshold, at the direction of their principal investigator, a run-in wash-out period of 6 weeks (42 days) is requested before they are eligible to return for a repeat visit 1. Patients who fail two urine N-acetyl-D-leucine tests (e.g., visit 1 and repeat visit 1) are ineligible for the study.

#### Randomization

At visit 2, patients will be randomized (1:1) to their respective sequence. Patients will be centrally randomized using Medpace’s ClinTrak Interactive Response Technology (IRT) web-based system. Medpace is responsible for the generation of the allocation sequence, and the IRT system will automatically assign participants to their respective sequence. The study utilizes a permuted block design for randomization. The LIVE randomization list is generated by an independent statistician based on the approved randomization plan. As the study is double-blinded, patients, their families, the study team, and the sponsor will be blinded to the randomization scheme and the sequences to which the patient is assigned. The randomization list will not be available to any person involved in the conduct of the study or the evaluation of the trial until the trial database is locked. The bioanalytical laboratory staff are authorized to receive the randomization list prior to the study conclusion to determine which samples should be analyzed according to standard operating procedures. As the trial is a crossover design, in which each patient will receive IB1001 and placebo, and serve as their own control, no stratification by age is performed.

#### Intervention periods

**Fig. 1:**
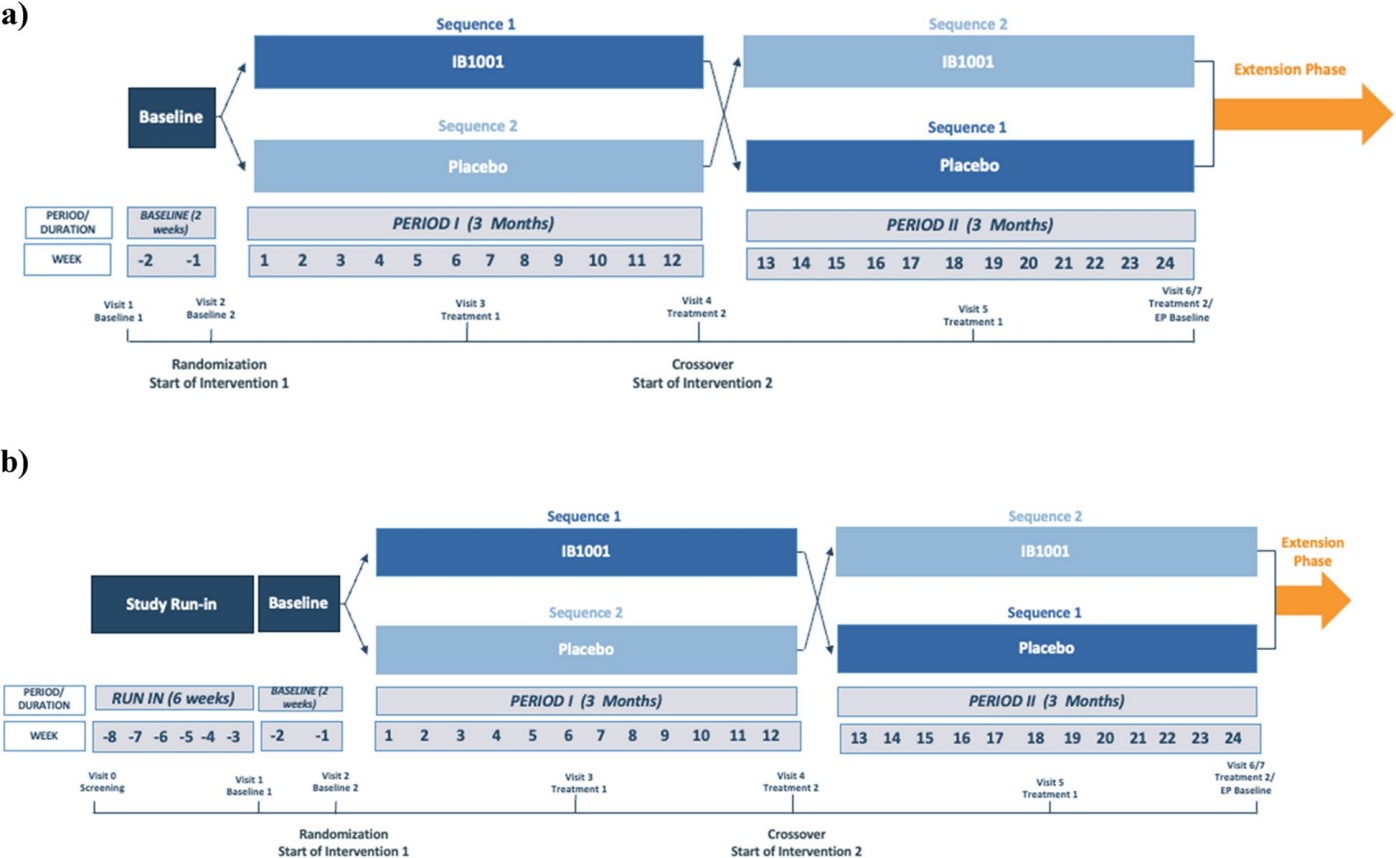
Parent Study schema. **a** Naïve patients screening pathway: patients who have not used any prohibited medications within 42 days of screening are “naïve.” Their initial screening visit is treated as visit 1 (baseline 1). **b** Non-naïve patient screening pathway: patients who have used or are unable to confirm or deny if they have used, any prohibited medication within the past 42 days are “non-naïve.” Patient will be given the opportunity to undergo a minimum of 42 days washout before returning for a repeat visit 1 (baseline 1). From visit 2 onward, the visit schedule is the same for naïve and non-naïve patients

Figure 1 displays the naïve and non-naïve study schemes for the Parent Study. Suppl. Tables 1 lists the schedule of enrolment and assessments together with pre-planned time points for clinic visits. Patients who have completed Visit 6 of the Parent Study have the opportunity to continue treatment with N-acetyl-L-leucine (IB1001) in an Extension Phase if the principal investigator determines it is in their best interest. The Extension Phase consists of a 1-year (351–379 day) treatment period followed by a 6 weeks (42–56 days) washout period. Table 2 lists the inclusion criteria for the Extension Phase.

**Table 2:** Inclusion criteria for patient participation in the Extension Study

Inclusion criteria
1. Completed visit 6 of the Parent Study
2. The principal investigator determines further treatment with IB1001 to be in patient’s best interest
3. Written informed consent signed by the patient and/or their legal representative/parent/impartial witness for participation in the Extension Phase
4. Patients are willing to continue to remain without the following prohibited medication from **visit 6** throughout the duration the Extension Phase**:** e) N-acetyl-DL-leucine (e.g., Tanganil®); f) N-acetyl-L-leucine (prohibited if not provided as investigational medicinal product [IMP]); g) Sulfasalazine; h) Rosuvastatin

**Fig. 2:**
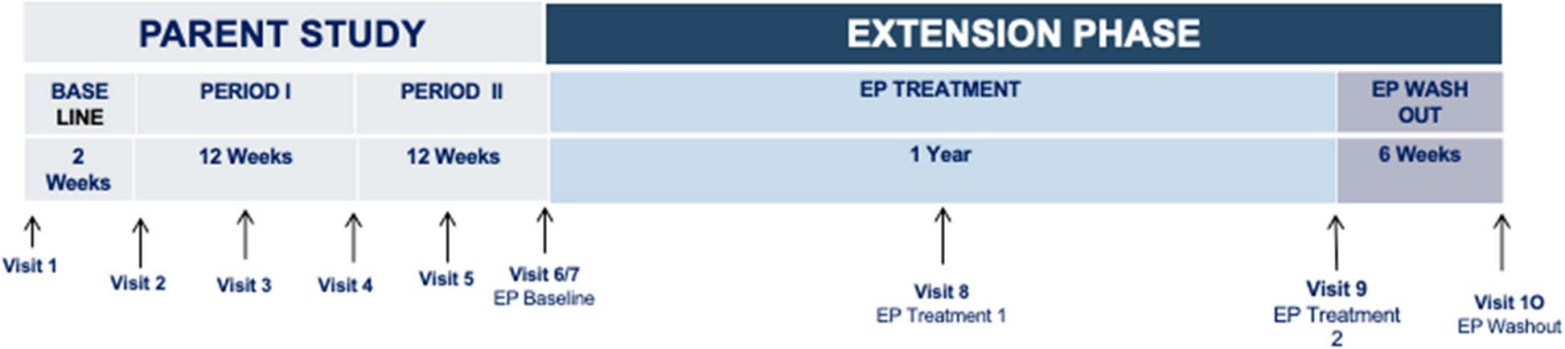
Extension Phase schema. Patients will be assessed approximately 4 times over a 64-week period: at the start of the Extension Phase, after 6 months of treatment, 1 year of treatment, and after a 42-day (+ 14 day) post-extension-phase treatment washout

Figure 2 displays the Extension Phase study schema. Suppl. Table 2 lists the schedule of enrolment and assessments together with pre-planned time points for clinic visits in the Extension Phase.

### Study drug

The dosage form of N-acetyl-L-leucine or the matching placebo is granules for oral suspension in a sachet (manufactured by Patheon France S.A.S., Bourgoin Facility, France) which are suspended in 40 mL water, orange juice, or almond milk. A marked measuring cup is provided to each patient.

### Administration and study drug dosage

During the treatment periods for both treatment sequences, the dosing of the study drug is as follows: patients aged ≥ 13 years or aged 4–12 years weighing ≥ 35 kg will take 4 g/day (2 g in the morning, 1 g in the afternoon, and 1 g in the evening). Patients aged 4–12 years weighing 25 to < 35 kg will take 3 g/day (1 g in the morning, 1 g in the afternoon, and 1 g in the evening). Patients aged 4–12 years weighing 15 to < 25 kg will take 2 g/day (1 g in the morning and 1 g in the evening). Medication should be taken at least 30 min before or at least 2 h after a meal. Compliance will be assessed upon a review of the inventory of IB1001 sachets returned by patients.

### Study objectives

The Parent Study and Extension Phase enable the investigation of both the symptomatic (12-week) and long-term (1-year) safety and efficacy of treatment with N-acetyl-L-leucine. The primary objective of the Parent Study is to evaluate the efficacy of N-acetyl-L-leucine versus placebo based on the Scale for the Assessment and Rating of Ataxia (SARA) or modified SARA (mSARA). In the Extension Phase, the primary objective is to evaluate the effects of N-acetyl-L-leucine based on the modified (5-domain) Niemann-Pick disease type C Clinical Severity Scale (NPC-CSS).

For both study phases, the secondary objectives are:

- To assess the clinical efficacy (symptomatic and long-term) of N-acetyl-L-Leucine on symptoms of ataxia, functioning, and quality of life for patients with NPC
- To evaluate the safety and tolerability of N-acetyl-L-leucine at 4 g/day in patients with NPC aged ≥ 4 years and older
- Extension Phase only: characterize the pharmacokinetics of N-acetyl-L-leucine in NPC patients

### Safety and efficacy parameters

#### Primary efficacy endpoint

The original SARA scale is an eight-item clinical rating scale (range 0–40, where 0 is the best neurological status and 40 is the worst). It is a reliable and valid clinical scale with a high internal consistency that measures the severity of symptoms and ataxia and increases with disease stage [16].

The original unmodified SARA was selected as the primary endpoint based on advice from EU National Regulatory Authorities (including Germany, Portugal, Spain, and the Netherlands) and the UK Medicines and Healthcare product Regulatory Authority. In the USA, the primary endpoint is the modified SARA (mSARA). This modification was based on the explicit advice of the US Food and Drug Administration, which requested the instrument be modified to:

- Include the domains that are the most clinically meaningful towards understanding the functional aspects of ataxia/symptoms in NPC patients
- Remove domains where there is no/little movement and which may impact the interpretation and power of the tool (i.e., improve the reliability and sensitivity of the instrument)

Accordingly, the mSARA was developed as a six-item clinical rating scale consisting of the original Gait, Speech Disturbance, Finger Chase, Nose-Finger test, Fast Alternating Hand Movement, and Heel-shin slide domains. It ranges from a score of 0 to 30, where 0 is the best neurological status and 30 is the worst.

The SARA scale will be the basis for the primary efficacy estimand. The mSARA scale will be considered as a supplementary estimand. Withdrawal from study medication due to adverse events and the taking of prohibited medication will be considered as intercurrent events (ICEs) and a treatment policy strategy will be considered for these ICEs. Withdrawal or early termination from the study for unspecified reasons and lost to follow-up will not be considered as ICEs, and the subsequent data will be considered as “missing.” Should these events occur, a last observation carried forward approach will be adopted based on observations at visits 2, 3 for period 1 and visits 4, 5 for period 2.

The model for analysis will be an analysis of covariance model with the difference in the SARA scores at the end of periods I and II as the dependent variable and SARA at baseline (visit 2) as the independent variable together with an indicator variable for sequence [17]. The estimated coefficient of the indicator for sequence will provide the least squares estimate of the difference in the treatment means on division by 2. Secondary endpoints will measure other symptoms and evaluate quality of life (Spinocerebellar Ataxia Functional Index (SCAFI) [18]; Modified Disability Rating Scale (mDRS) [19, 20]; Quality of Life EQ-5D-5L for patients aged ≥ 18; EQ-5D-Y for patients aged < 18 years [21]; and Investigator, Caregiver, Patient Clinical Global Impression Scale (CGI)) [22]. Descriptive statistics will be provided for these measures at each visit, and select secondary endpoints will be evaluated statistically based on a comparison of the period differences (period II–period I) between the two treatment sequences in an analysis of covariance (ANCOVA) model with terms for baseline and treatment sequence.

#### Safety parameters

Adverse events (serious and non-serious), concomitant drug and non-drug therapies, safety laboratory blood samples (hemoglobin, erythrocytes, hematocrit, thrombocytes, leukocytes, sodium, potassium, urea, creatinine, serum bilirubin level, aspartate aminotransferase, alanine aminotransferase, alkaline phosphatase, lactate dehydrogenase, follicle-stimulating hormone for postmenopausal women only), and urine samples (leukocytes, nitrite, urobilinogen, protein, pH, occult blood (erythrocytes, leucocytes), specific gravity, ketones, bilirubin, glucose) will be collected routinely throughout the study. Sparse pharmacokinetic blood sampling will be conducted in the Parent Study (visit 1–visit 6). Blood samples for the quantification of N-acetyl-L-leucine in plasma will be obtained at visit 7 and visit 9. Urine samples will also be collected for measuring concentrations of N-acetyl-D-leucine at the time points designated on the schedule of events (Suppl. Tables 1 and 2). At visit 1, this urine sample serves as a key enrollment criterion testing for the use of the prohibited medication N-acetyl-DL-leucine. Vital signs, physical exams, height/weight, and electrocardiograms will also be collected at the time points designated on the schedule of events (Suppl. Tables 1 and 2). A detailed description of the safety parameters is provided in Suppl. Material I.

All statistical analyses will be detailed in a separate statistical analysis plan. For each of the primary and secondary endpoints, there will be evaluations within key subgroups: naïve versus non-naïve as determined at screening; age (pediatric versus adult at the time of enrollment); age/weight/dosing group; age of diagnosis: early-infantile (< 2 years), late-infantile (2 to < 6 years), juvenile (6 to < 15 years), adolescent/adult (≥ 15 years); disease severity based on SARA below/above the median SARA score at visit 1; gender (male versus female); region (US versus rest of world); patients taking miglustat versus patients not taking miglustat; intra-patient variability between SARA score at visit 1 (baseline 1) versus visit 2 (baseline 2) (below/above median). These evaluations will be based on plotting treatment differences together with 90% confidence intervals within each subgroup.

In a one-sided test at the *p* = 0.05 level, a total sample size of 46 in a two-treatment, two-period placebo-controlled cross-over trial achieves approximately 80% power for treatment comparisons in relation to the SARA/mSARA total score assuming a true mean difference of 1.0/0.85 (respectively) and a standard deviation for the total SARA/mSARA score between 7.5 and 8.5 and 6.375 and 7.225 (respectively) based on an analysis of covariance with the baseline SARA/mSARA score at the start of period I as the covariate. These results, based on extensive simulations, assume a correlation of 0.95 between each of the pairwise outcomes: baseline for period I, endpoint for period I, and endpoint for period II. The values for the standard deviation and the correlations used in the calculation are guided by the results of the IB1001-201 study. These levels of power are maintained when there is a positive or negative period effect of up to 0.5 and 0.425 units on the SARA/mSARA scale, respectively.

A description of data collection is provided in Suppl. Material II.

## Discussion

Given the lack of global symptomatic or disease-modifying therapies for NPC and other lysosomal diseases, there is an urgent need for effective and well-tolerated drug treatments. This multi-center, multinational randomized double- blind placebo-controlled crossover phase III trial was designed through a collaboration between national regulatory agencies, leading clinical experts, patient organizations, and the industry sponsor. This design addresses the unique ethical and practical challenges of conducting clinical trials for these orphan, heterogeneous patient populations in order to better capture N-acetyl-L-leucine’s therapeutic effect and thus expedite the development and availability of this promising drug candidate [23].

## Trial status

At the time of manuscript submission, the protocols for IB1001-301 (US V1.2 07-Jan-2022; Global: V2.0, 08-Mar-2022; Swiss V2.1 14-Jul-2022) have been accepted/approved in each country where the study will be conducted, including the US Food and Drug Administrations, UK Medicines and Healthcare products Regulatory Authority, German Federal Institute for Drugs and Medical Devices, Slovakia Štátny ústav pre kontrolu liečiv, Switzerland Swissmedic, Netherlands Central Committee on Research Involving Human Subjects, Czech Republic State Institute for Drug Control, and Australia Therapeutic Goods Administration as well as respective research ethics committees (REC)/institutional review boards (IRB) (active approved protocol version varies based on status of enrollment per site/per study). The first study participant was enrolled on 30 June 2022. Recruitment is ongoing and expected to complete December 2022.

## Supplementary Information

**Additional file 1: Supplementary Material I.** Safety Parameters. **Supplementary Material II.** Data Collection.

**Additional file 2: Supplementary Table 1.** Parent Study schedule of enrolment, interventions, and assessments. **Supplementary Table 2.** Extension Phase schedule of enrolment, interventions, and assessments.

**Additional file 3. **SPIRIT Checklist for Trials.

## Data Availability

All investigators will be provided with full access to all the final data set in the study. Data sharing is not applicable to this article as no datasets were generated or analyzed during the current studies (study protocols).

## Abbreviations

ANCOVA: Analysis of covariance
CGI: Clinical Global Impression
DSMB: Data safety monitoring board
EP: Extension Phase
GM2: GM2 gangliosidoses
ICE: Intercurrent events
IRB: Institutional review boards
IRT: Interactive Response Technology
mDRS: Modified Disability Rating Scale
mSARA: Modified Scale for the Assessment and Rating of Ataxia
NPC: Niemann-Pick type C
NPC-CSS: NPC clinical severity scale
PI: Principal investigator
REC: Research ethics committees
SARA: Scale for the Assessment and Rating of Ataxia
SCAFI: Spinocerebellar Ataxia Functional Index
