# Supplementary material for: N-acetyl-L-leucine for Niemann-Pick type C: a multinational double-blind randomized placebo-controlled crossover study
Source: Trials. 2023 May 29;24:361. doi: 10.1186/s13063-023-07399-6 (PMC10226221; doi:10.1186/s13063-023-07399-6)
Supplement: Supplementary file 1 — Additional file 1: Supplementary Material I. Safety Parameters. Supplementary Material II. Data Collection. [file 13063_2023_7399_MOESM1_ESM.docx]

**Supplementary Material I: Safety Parameters**

**Laboratory examinations - blood**

A routine blood sample will be taken to exclude liver or kidney failure, and a pregnancy test for women of childbearing potential will be performed. The following laboratory parameters will be assessed at each visit (except Visit 0) via blood draw: hemoglobin, erythrocytes, hematocrit, thrombocytes, leukocytes, sodium, lactate dehydrogenase, potassium, creatinine, serum bilirubin level, aspartate aminotransferase, alanine aminotransferase, urea, alkaline phosphatase, follicle-stimulating hormone (for postmenopausal women only). A plasma pregnancy test will be performed at Visit 1 for females of childbearing potential.

Sparse Pharmacokinetic blood sampling will be conducted during the initial treatment sequence Visit 2, 4, and 6. Blood samples for the quantification of N-acetyl-L-leucine in plasma will be obtained at Visit 7 and Visit 9. The first sample will be taken before first /last dosing of N-acetyl-L-leucine in the Extension Phase; subsequent samples will be taken at 30 minutes (+/- 5 minutes), 60 minutes (+/- 5 minutes), 90 minutes (+/- 10 minutes), 120 minutes (+/- 10 minutes), 150 (+/- 15 minutes), 180 minutes (+/- 15 minutes), 240 minutes (+/- 15 minutes), and 360 minutes (+/- 15 minutes) after the first/last extension phase IB1001 dose.

The total amount of blood taken per subject during the first treatment sequence (Visit 1 – Visit 6) will be 78 mL (42 mL blood for the safety analyses, 24 mL blood for the Pharmacokinetic analyses, 12 mL for research purposes). The total amount of blood taken per subject during the Extension phase treatment sequence is 97 – 104 mL (21 mL – 28 mL (depending on the date of Visit 7) for safety analysis, and 72 mL for the Pharmacokinetic analysis, and 4 mL for research purposes).

**Laboratory examinations – urine**

The following laboratory parameters will be assessed via urine at each visit (except Visit 0): Leukocytes, Nitrite, Urobilinogen, Protein, pH, Occult blood (erythrocytes, leucocytes), Specific gravity, Ketones, Bilirubin, Glucose.

Urine samples will also be collected for concentrations of N-acetyl-D-leucine at every visit. At Visit 1, this Urine sample score serves as a key enrollment criterion testing for the use of the prohibited medication N-acetyl-DL-leucine.

**Height, Weight, Vital Signs, Physical Exam**

Patient’s height, weight, systolic/diastolic blood pressure and pulse will be measured routinely throughout the duration of the study according to the schedule of events (Suppl. Table 1 and 2).

A complete physical examination that is limited to the following body systems will be conducted according to the schedule of events (Suppl. Table 1 and 2): general appearance (including skin), head and neck, eyes and ears, nose and throat, pulmonary, cardiovascular, gastrointestinal, musculoskeletal, lymphatic, and neurological.

### Electrocardiograms

12-lead electrocardiograms will be performed at the time points designated on the schedule of events (Suppl. Table 1 and 2) and include the following measurements: Corrected QT, and whether the electrocardiograms is Normal, Abnormal not clinically significant, or Abnormal clinically significant for the patient. Intervals of the electrocardiograms will be recorded in the electronic case report form. Electrocardiograms may be performed at other visits at the discretion of the Investigator.

**Concomitant drug and non-drug therapies**

Trial participants should not begin physiotherapy or speech therapy while they are enrolled in the trial. If they are already under therapy, the number of sessions of physiotherapy and speech therapy (measured in hours of therapy per week) will be documented in the patient’s medical record and in the electronic case report form during the trial and for 6 weeks prior to screening. To adhere to the protocol, the non-pharmacological concomitant therapy should be consistent 6-weeks before Visit 1 and continued with the same intensity while the patient is enrolled in the trial.

Guided by the eligibility criteria, the administration variants of the IMP, -Acetyl-DL-Leucine (e.g. Tanganil^®^) or N-acetyl-L-leucine (if not provided as IMP) are not allowed during the trial. Sulfasalazine and Rosuvastatin are not allowed because drug interactions with substrates of transporters MDR1, BCRP, or BSEP cannot be excluded as N-acetyl-L-leucine is an inhibitor of these transporters *in vitro.*

**Unblinding**

Unblinding by request of an Investigator should occur only in the event of an emergency or adverse event for which it is necessary to know the study treatment to determine an appropriate course of therapy for the patient. If the Investigator must identify the treatment assignment of an individual patient, the Investigator or qualified designee should request the medication information from the centralized randomization system. They should not attempt to get this information from the site’s unblinded pharmacist or qualified designee. The documentation received from the centralized randomization system indicating the code break must be retained with the patient’s source documents in a secure manner so as not to un-blind the treatment assignment to other Site or Sponsor personnel. The Investigator is also advised not to reveal the study treatment assignment to other site or Sponsor personnel.

Prior to unblinding, and if the situation allows, the Investigator should try to contact the site monitor or the Sponsor’s Medical Monitor in order to get additional information about the study drug. If this is impractical, the Investigator must notify the site monitor or the Sponsor’s Medical Monitor as soon as possible, without revealing the treatment assignment of the unblinded patient. The Investigator must document the patient identification and the date and time for breaking the blind and must clearly explain the reasons for breaking the code.

**Participation discontinuation**

Patients may withdraw from the study at any time at their own request without stating the reason(s) for withdrawal. The Principal Investigator may decide that a patient should be withdrawn from the study or from the study drug. Patients who discontinue study drug prior to completing the full treatment period will be asked to complete the remaining study visits as far as possible and complete safety assessments at a minimum. If unwilling to complete the remaining study visits, regardless of the reason for withdrawal, best efforts should be made to have the patient take part in early termination procedures, preferably 7-14 days following the last dose of the study drug, unless the patient is lost to follow-up or has withdrawn his/her consent to further study participation. The reason for withdrawal (if available) will be recorded in the electronic case report form.

**Safety assessment**

The Principal Investigator is responsible for monitoring the safety of patients who have been enrolled in this study and for accurately documenting and reporting information as described in this section.

In addition, the investigator will monitor the degree of stress to patients and the risk threshold throughout the trial.

Patients will be instructed to report to the Principal Investigator any Adverse Events that they experience. The Principal Investigator will ask about the occurrence of Adverse Events at each visit. Investigators are required to document all Adverse Events occurring during the clinical study, commencing with the signing of the informed consent form through the End of Study Visit (for patients who continue into the Extension Phase, Visit 10 for patients 42 days after the last dose with IB1001. Patients who do not continue in the Extension Phase will have a follow-up phone call within 14 days of Visit 6). Adverse event recording will continue for patients who discontinue study treatment but remain on-study, until their early termination Visit has been completed. The Principal Investigator will judge the intensity (mild, moderate, or severe), seriousness, and causality (not related or related) of all adverse events.

All adverse events will be listed by trial site and patient and displayed in summary tables that provide data combined across all sites. The incidence of adverse events and their relationship to the study drug will be analyzed descriptively, guided by the Medical Dictionary for Regulatory Activities classification.

**Supplementary Material II: Data collection**

Study data for all patients will be collected in a confidential fashion using an electronic case report form supported by Medpace’s Clintrak EDC. Access to the electronic case report form is restricted to staff members not involved in any aspect of the blinded evaluations. All the information required by the protocol must be documented and any omissions explained. The Investigator must review all electronic case report form entries for completeness and accuracy. Source documents, including all demographic and medical information, electronic case report form and informed consent form for each patient in the study must be maintained by the Investigator. All information in the electronic case report form must be traceable to the original source documents. An audit trail of all changes to this database, including the date, reason for the data change and who made the change, will be maintained within the same database. The audit trail will be part of the archived data at the end of the study. Concomitant medication and adverse events will be coded using standardized medical dictionaries. Data management procedures are defined in the Data Management Plan.
