# Supplementary material for: N-acetyl-L-leucine for Niemann-Pick type C: a multinational double-blind randomized placebo-controlled crossover study
Source: Trials. 2023 May 29;24:361. doi: 10.1186/s13063-023-07399-6 (PMC10226221; doi:10.1186/s13063-023-07399-6)
Supplement: Supplementary file 2 — Additional file 2: Supplementary Table 1. Parent Study schedule of enrolment, interventions, and assessments. Supplementary Table 2. Extension Phase schedule of enrolment, interventions, and assessments. [file 13063_2023_7399_MOESM2_ESM.docx]

**Supplementary Table 1**

|  | *Non-Naïve only* | *Non-Naïve only* |  |  |  |  |  |  |  |  |
| --- | --- | --- | --- | --- | --- | --- | --- | --- | --- | --- |
| Patient information and informed consent process | X |  | X^1^ |  |  |  |  |  |  |  |
| Inclusion / exclusion criteria | X |  | X^1^ |  |  |  |  | X |  |  |
| Patient demographics (in accordance with local regulations) | X |  | X^1^ |  |  |  |  |  |  |  |
| Patient weight (only) | X |  |  |  |  |  |  |  |  |  |
| Vital signs | X |  | X | X | X | X | X | X |  | X |
| Physical Examination (including weight) |  |  |  | X |  | X |  | X |  | X |
| Confirmation prohibited medications have not been used in the past 42 days at Visit 1/since Visit 1 for Visit 2 |  |  | X | X |  |  |  |  |  |  |
| Confirmation prohibited medications have been used within past 42 days / classify patient as “non-naïve” | X |  |  |  |  |  |  |  |  |  |
| Blinded Randomization |  |  |  | X |  |  |  |  |  |  |
| Relevant medical history | X |  | X^1^ |  |  |  |  |  |  |  |
| 60-Day drug history | X |  | X^1^ |  |  |  |  |  |  |  |
| Medication History: Confirm if N-Acetyl-Leucine ever used | X |  | X^1^ |  |  |  |  |  |  |  |

| Documentation of therapy / concomitant medication | X |  | X | X | X | X | X | X | X | X |
| --- | --- | --- | --- | --- | --- | --- | --- | --- | --- | --- |
| 12-lead electrocardiogram (ECG) |  |  | X |  | X |  | X |  |  | X |
| Urine Test for N-Acetyl-D-Leucine |  |  | X^7^ | X | X | X | X | X |  | X |
| Blood safety laboratory tests |  |  | X | X | X | X | X | X |  | X |
| Blood draw for sparse PK |  |  |  | X |  | X |  | X |  | X |
| Blood draw for research purposes |  |  |  | X |  | X |  | X |  | X |
| Follicle stimulating hormone serum |  |  | X |  |  |  |  |  |  |  |
| Urinalysis |  |  | X | X | X | X | X | X |  | X |
| Serum bHCG/pregnancy (if applicable) |  |  | X |  |  |  |  |  |  |  |
| Urine by dipstick for pregnancy test |  |  |  | X | X | X | X | X |  | X |

| Quality of Life EQ-5D-5L for patients aged ≥18 years; EQ-5D-Y for children aged <18 years |  |  | X | X | X | X | X | X |  | X |
| --- | --- | --- | --- | --- | --- | --- | --- | --- | --- | --- |
| Niemann-Pick type C Clinical Severity Scale (NPC-CSS) |  |  |  | X |  | X |  | X |  | X |
| Scale for Ataxia Rating (SARA) | X |  | X | X | X | X | X | X |  | X |
| Modified Disabling Rating Score (mDRS) |  |  | X | X | X | X | X | X |  | X |
| Scale for Spinocerebellar Ataxia Functional Index (SCAFI) | X |  | X | X | X | X | X | X |  | X |
| Clinical Global Impression of Severity (CGI-S) by Physician / Caregiver/ Patient |  |  | X | X | X | X | X | X |  | X |
| Clinical Global Impression of Change (CGI-I) by Physician / Caregiver/ Patient |  |  |  |  |  | X |  | X |  | X |
| Exit Interview |  |  |  |  |  |  |  | X |  |  |
| Documentation of AEs | X |  | X | X | X | X | X | X | X | X |
| Dispensing of study drug |  |  |  | X | X^15^ | X | X^15^ |  |  |  |
| Intake of study drug at site |  |  |  | X^16^ |  | X |  |  |  |  |
| Return of study drug |  |  |  |  | X | X | X | X |  | X |
| Study drug compliance check |  |  |  |  | X | X | X | X |  | X |

1 Naïve patients only

**Supplementary Table 2**

| Patient information and informed consent process | X |  |  |  |  |  |  |
| --- | --- | --- | --- | --- | --- | --- | --- |
| Inclusion / exclusion criteria | X |  |  |  |  |  |  |
| Physical Examination (+weight) | X |  | X |  | X | X | X |
| Documentation of concomitant medication | X |  | X |  | X | X | X |
| Documentation of frequency of therapy (hours per week) / concomitant medications | X |  | X |  | X | X | X |
| Vital signs | X |  | X |  | X | X | X |
| 12-lead electrocardiogram (ECG) |  |  |  |  | X |  | X |
| Blood safety laboratory tests | X |  | X |  | X | X | X |
| PK Blood Sampling |  | X |  |  | X |  |  |
| Blood Draw for Research Purposes | X |  |  |  | X |  | X |
| Urinalysis | X |  | X |  | X | X | X |
| Urine by dipstick for pregnancy test | X |  | X |  | X | X | X |
| Urine test for N-Acetyl-D-Leucine | X |  |  |  |  | X | X |
| Quality of Life EQ-5D-5L for patients aged ≥18; EQ-5D-Y for children aged <18 years | X |  | X | X |  | X | X |
| Scale for Ataxia Rating (SARA) | X |  | X | X |  | X | X |
| Niemann-Pick Disease type C Clinical Severity Scale | X |  | X | X |  | X | X |
| Clinical Global Impression of Severity (CGI-S) by Physician / Caregiver (if applicable)/ Patient (if able) | X |  | X | X |  | X | X |
| Documentation of AEs | X | X | X | X | X | X | X |
| Dispensing of study drug |  | X | X |  |  |  |  |
| Intake of study drug at site |  | X |  |  | X |  |  |
| Return of study drug |  |  | X |  | X |  | X |
| Study drug compliance check |  |  | X |  | X |  | X |
